# A data-driven approach to predicting diabetes and cardiovascular disease with machine learning

**DOI:** 10.1186/s12911-019-0918-5

**Published:** 2019-11-06

**Authors:** An Dinh, Stacey Miertschin, Amber Young, Somya D. Mohanty

**Affiliations:** 1 Department of Mathematics and Computer Science, Eastern Oregon University, La Grande, OR USA; 2 Department of Mathematics and Statistics, Winona State University, Winona, MN USA; 3 Department of Statistics, Purdue University, West Lafayette, IN USA; 4 Department of Computer Science, University of North Carolina at Greensboro, Greensboro, NC USA

**Keywords:** Machine learning, Health analytics, Ensemble learning, Feature learning

## Abstract

**Background:**

Diabetes and cardiovascular disease are two of the main causes of death in the United States. Identifying and predicting these diseases in patients is the first step towards stopping their progression. We evaluate the capabilities of machine learning models in detecting at-risk patients using survey data (and laboratory results), and identify key variables within the data contributing to these diseases among the patients.

**Methods:**

Our research explores data-driven approaches which utilize supervised machine learning models to identify patients with such diseases. Using the National Health and Nutrition Examination Survey (NHANES) dataset, we conduct an exhaustive search of all available feature variables within the data to develop models for cardiovascular, prediabetes, and diabetes detection. Using different time-frames and feature sets for the data (based on laboratory data), multiple machine learning models (logistic regression, support vector machines, random forest, and gradient boosting) were evaluated on their classification performance. The models were then combined to develop a weighted ensemble model, capable of leveraging the performance of the disparate models to improve detection accuracy. Information gain of tree-based models was used to identify the key variables within the patient data that contributed to the detection of at-risk patients in each of the diseases classes by the data-learned models.

**Results:**

The developed ensemble model for cardiovascular disease (based on 131 variables) achieved an Area Under - Receiver Operating Characteristics (AU-ROC) score of 83.1% using no laboratory results, and 83.9% accuracy with laboratory results. In diabetes classification (based on 123 variables), eXtreme Gradient Boost (XGBoost) model achieved an AU-ROC score of 86.2% (without laboratory data) and 95.7% (with laboratory data). For pre-diabetic patients, the ensemble model had the top AU-ROC score of 73.7% (without laboratory data), and for laboratory based data XGBoost performed the best at 84.4%. Top five predictors in diabetes patients were 1) waist size, 2) age, 3) self-reported weight, 4) leg length, and 5) sodium intake. For cardiovascular diseases the models identified 1) age, 2) systolic blood pressure, 3) self-reported weight, 4) occurrence of chest pain, and 5) diastolic blood pressure as key contributors.

**Conclusion:**

We conclude machine learned models based on survey questionnaire can provide an automated identification mechanism for patients at risk of diabetes and cardiovascular diseases. We also identify key contributors to the prediction, which can be further explored for their implications on electronic health records.

## Background

Diabetes and Cardiovascular disease (CVD) are two of the most prevalent chronic diseases that lead to death in the United States. In 2015, about 9% of the U.S. population had been diagnosed with diabetes while another 3% were undiagnosed. Furthermore, about 34% had prediabetes. However, of those adults with prediabetes almost 90% of them were unaware of their condition [1]. CVD on the other hand is the leading cause of one in four deaths every year in the U.S. [2]. Approximately, 92.1 million American adults are living with some form of CVD or the after-effects of stroke, where the direct and indirect costs of healthcare is estimated to be more than $329.7 [3]. Additionally, there is a correlation between CVD and diabetes. American Heart Association reports at least 68% of people age 65 or older with diabetes, die of heart disease [4]. A systematic literature review by Einarson et al. [5], the authors concluded that 32.2% of all patients with type 2 diabetes are affected by heart disease.

In the world of ever-growing data where hospitals are slowly adopting big data systems [6], there are great benefits to employing data analytics in the health care system to provide insights, augment diagnosis, improve outcomes, and reduce costs [7]. In particular, successful implementation of machine learning enhances the work of medical experts and improves the efficiency of the health care system [8]. Significant improvements in diagnostic accuracy have been shown through the performance of machine learning models along with clinicians [9]. Machine learning models have since been used in the prediction of many common diseases [10, 11], including the prediction of diabetes [12, 13], detection of hypertension in diabetic patients [14], and classification of patients with CVD among diabetic patients [15].

Machine learning models can be useful in the identification of patients with diabetes or heart disease. There are often many factors that contribute to identifying patients who are at risk for these common diseases. Machine learning methods can help identify hidden patterns in these factors that may otherwise be missed.

In this paper, we use supervised machine learning models to predict diabetes and cardiovascular disease. Despite the known association between these diseases, we design the models to predict CVD and diabetes separately in order to benefit a wider range of patients. In turn, we are able to identify the feature commonalities between the diseases which affect their prediction. We also consider the prediction of prediabetes and undiagnosed diabetes. The National Health and Nutrition Examination Survey (NHANES) dataset is used to train and test multiple models for the prediction of these diseases. This paper also explores a weighted ensemble model which combines the results of multiple supervised learning models to increase prediction ability.

### NHANES Data

The National Health and Nutrition Examination Survey (NHANES) [16] is a program designed by the National Center for Health Statistics (NCHS), which is used to assess the health and nutritional status of the U.S. population. The dataset is unique in the aspect that it combines survey interviews with physical examinations and laboratory tests conducted at the medical locations. The survey data consists of socio-economic, demographic, dietary, and health-related questions. The laboratory tests consist of medical, dental, physical, and physiological measurements conducted by medical personnel.

The continuous NHANES data was initiated in 1999, and is ongoing with a sample each year consisting of 5000 participants. The sampling utilizes a nationally representative civilian sample identified though a multistage probability sampling design. Apart from the laboratory results of the individuals, prevalence of chronic conditions in the population is also collected. For example, information about anemia, cardiovascular disease, diabetes, environmental exposures, eye diseases, and hearing loss are collected.

NHANES provides insightful data that has made important contributions to people in the United States. It gives researchers important clues to the causes of disease based on the distribution of health problems and risk factors in the population. It also allows health planners and government agencies to detect and establish policies, plan research, and health promotion programs to improve present health status and prevent future health problems. For example, past surveys’ data is used to create growth charts to evaluate children’s growth, which have been adapted and adopted worldwide as a reference standard. Education and prevention programs increasing public awareness, emphasizing diet and exercise were intensified based on the indication of undiagnosed diabetes, overweight prevalence, hypertension and cholesterol level figures.

### Machine Learning Models

In our study, we utilize multiple supervised learning models for classification of at-risk patients. In supervised learning, the learning algorithm is provided with training data that contains both the recorded *observations* and the corresponding *labels* for the category of the observations. The algorithm uses this information to build a model that, when given new *observations*, can predict which output *label* should be associated with each new observation. In the following paragraphs, the models used in this project are briefly described.

- Logistic Regression is a statistical model that finds the coefficients of the best fitting linear model in order to describe the relationship between the logit transformation of a binary dependent variable, and one or more independent variables. This model is a simple approach to prediction which provides baseline accuracy scores for comparisons with other non-parametric machine learning models [17].
- Support Vector Machines (SVM) classify data by separating the classes with a boundary, i.e. a line or multi-dimensional hyperplane. Optimization ensures that the widest boundary separation of classes is achieved. While SVM often outperforms logistic regression, the computational complexity of the model results in long training durations for model development [18].
- Ensemble models synthesize the results of multiple learning algorithms to obtain better performance than individual algorithms. If used correctly, they help decrease variance and bias, as well as improve predictions. Three ensemble models used in our study were random forests, gradient boosting, and a weighted ensemble model.
  - Random Forest Classifier (RFC) is an ensemble model that develops multiple random decision trees through a bagging method [19]. Each tree is an analysis diagram that depicts possible outcomes. The average prediction among the trees is taken into account for global classification. This reduces drawback of large variance in decision trees. Decision splits are made based on impurity and information gain [20].
  - Gradient Boosted Trees (GBT) [21] is also an ensemble prediction model based on decision trees. In contrast to Random Forest, this model successively builds decision trees using gradient descent in order to minimize a loss function. A final prediction is made using a weighted majority vote of all of the decision trees. We consider an implementation of gradient boosting, XGBoost [22], which is optimized for speed and performance.
  - A Weighted Ensemble Model (WEM) that combines the results of all aforementioned models was also used in our analysis. The model allows multiple predictions from disparate models to be averaged with weights based on a individual model’s performance. The intuition behind the model is the weighted ensemble could potentially benefit from the strengths of multiple models in order to produce more accurate results.

Based on the prior research [12, 13] in the domain, Logistic regression and SVM models were chosen as the performance baseline models for our study. RFC, GBT, and WEM based models were developed within our study in order to take advantage of non-linear relationships which may exist within the data for disease prediction. The study chose to exclude neural networks from its analysis due to the “black-box” (non-transparency) nature of the approach [23].

## Methods

**Fig. 1:**
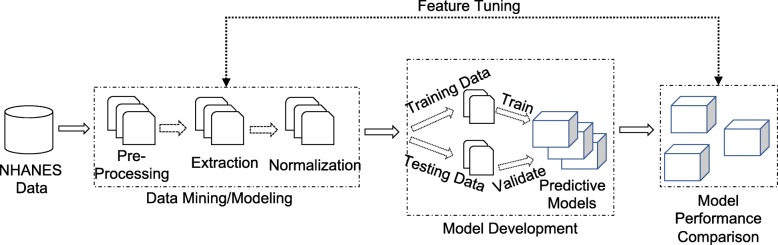
Model Development and Evaluation Pipeline. A flow chart visualizing the data processing and model development process

Figure 1 depicts the flow from raw data through the development of predictive models, and their evaluation pipeline towards identifying risk probabilities of diabetes or cardiovascular disease in subjects. The pipeline consists of three distinct stages of operation: 1) Data mining and modeling, 2) Model development, and 3) Model evaluation.

### Data Mining and Modeling

#### Dataset Preprocessing

The first stage of the pipeline involves data mining methods and techniques for converting raw patient records to an acceptable format for training and testing machine learning models. In this stage, the raw data of patients was extracted from the NHANES database to be represented as records in the preprocessing step. The preprocessing stage also converted any undecipherable values (errors in datatypes and standard formatting) from the database to null representations.

The patient records were then represented as a data frame of features and a class label in the feature extraction step. The features are an array of patient information collected via the laboratory, demographic, and survey methods. The class label is a categorical variable which will be represented as a binary classification of the patients: 0 - Non-cases, 1 - Cases. Categorical features were encoded with numerical values for analysis. Normalization was performed on the data using the following standardization model: $x' = \frac {x-\bar {x}}{\sigma }$, where *x* is the original feature vector, $\bar x$ is the mean of that feature vector, and *σ* is its standard deviation.

Previous attempts to predict diabetes with machine learning models using NHANES data, put forth a list of important variables [12, 13]. In the work done by Yu et al. [13], the authors identified fourteen important variables — family history, age, gender, race and ethnicity, weight, height, waist circumference, BMI, hypertension, physical activity, smoking, alcohol use, education, and household income, for training their machine learning models. The feature selection was based based on methods of combining SVMs with feature selection strategies as described in Chen et al. [24]. Semerdjian et al. [12] chose the same features as Yu et al. and added two more variables — cholesterol and leg length. The features were based on the analysis done by Langner et al. [25], where they used genetic algorithms and tree based classification of identification of key features for diabetes prediction.

With a goal to develop a data-driven model, all possible variables were extracted from the raw NHANES dataset for the preliminary features. The data was then examined for continuity and availability of each variable across the specific categories and years. This was important because of the underlying NHANES data structure, where each biennial cycle was split into multiple datasets based on the category of the variable. The analysis showed missing data was a result of data recorded by questions conditioned on responses to prior questions (such as age, gender, or pregnancy status). Furthermore, some discontinuity of variables was due to inconsistent data collection by NHANES across different cycles. Some variables were simply given different names in different cycles. Based on the manual analysis, re-coding of some variable names were performed. After performing the aforementioned steps on the raw dataset, only 189 variables out of approximately 3900 variables from the NHANES database were continuous across all cycles from 1999 to 2014. The data was further analyzed for missing values within the variables, and any with more than 50% of missing values were dropped from the dataset. This lead to a further reduction in number of available variables to 123 for the 1999-2014 cycle.

For *diabetes dataset*, two different datasets were created based on the variable utilization cycles. This was done in order to maximize variable availability across different timeframes and to study its effect on machine learning models. The first dataset is based on the original timeframe of 1999-2014 consisting of 123 variables, while the second dataset had a timeframe of 2003-2014 with 168 variables.

For *CVD dataset*, a timeframe of 2007-2014 was used, which maximized the number of available variables to 131. Specifically, the dataset included the physical activity variables which are considered important factors of cardiovascular disease [26].

Each dataset, was further categorized into laboratory (contains laboratory results) versus no laboratory (survey data only) datasets. Laboratory results were any feature variables within the dataset that were obtained via blood or urine tests. Recategorization of the data into these groups enables performance analysis of machine learning models in cases where laboratory results are unavailable for patients, which facilitates the detection of at-risk patients based only on a survey questionnaire.

#### Subject Exclusion and Label Assignment

In our study, all datasets were limited to non-pregnant subjects and adults of at least twenty years of age. This allows us to focus in on the prediction of Type II Diabetes, and exclude other types such as gestational diabetes, which is exclusive to pregnant women, and Type I Diabetes, which usually develops in children and adolescents. This exclusion is consistent with the prior research conducted by Yu et al. [13] and Semerdjian et al. [12].

For *diabetes classification*, labels are assigned to the dataset under two different schemes 1) Diabetic and 2) Pre/ Undiagnosed Diabetic. This is similar to the classification schemes set up by Yu et al. [13]. In the first scheme, subjects were considered to have diabetes (*label*=1) if they answered “Yes” to the question “Have you ever been told by a doctor that you have diabetes?” or had a blood glucose level greater than or equal to 126 mg/dl, other subjects were considered not to have diabetes (*label*=0). The resulting classification scheme was named Case I.

The second classification scheme - Case II - was developed for predicting subjects with undiagnosed diabetes or pre-diabetes. Subjects were labeled undiagnosed diabetics (*label*=1) if they answered “No” to the question “Have you ever been told by a doctor that you have diabetes?” and had a blood glucose level greater than or equal to 126 mg/dl. Subjects were labeled pre-diabetic (*label*=1) if their blood glucose level was between 100 and 125 mg/dl. Diagnosed diabetics were excluded from Case II, and all other subjects were considered to be non-cases (*label*=0). Subjects who had a missing value for diabetes classification were excluded from the data for both cases.

For *CVD classification*, subjects were labeled as having the disease (*label*=1) if they answered “Yes” to the cardiovascular symptoms/conditions represented by the question: “Have you ever been told by a doctor that you had congestive heart failure, coronary heart disease, a heart attack, or a stroke?” If the subject answered “No” to all four conditions, then the subject was labeled as not having the disease (*label*=0). Note that these conditions are common indicators of cardiovascular disease [27].

Table 1 summarizes diabetes classification criteria, and the corresponding label assignment for each case is shown in Table 2. The classification and label assignments for CVD are summarized in Table 3. The classification schemes were applied to the three timeframes described previously in Section 4. This resulted in five separate datasets for classification: four for diabetes classification, and one for CVD classification. The timeframe, number of variables, observations, and number of cases (*label*=1) and non-cases (*label*=0) for each dataset are all summarized in Table 4.

**Table 1:** Diabetes classification criteria

Criteria		Classification
Answered “yes” to “Have you been told by a doctor that you have diabetes” ^*γ*^*or* had a Plasma Glucose ≥126 mg/dl ^*δ*^	⇒	Diabetic
Answered “no”, but had a Plasma Glucose ≥126 mg/dl	⇒	Undiagnosed diabetic
Had a Plasma Glucose between 100−125 mg/dl	⇒	Prediabetic
Had a Plasma Glucose ≤100 mg/dl	⇒	Not diabetic

^*γ*^NHANES survey questionnaire

^*δ*^NHANES laboratory results

**Table 2:** Label assignments for Case I and Case II

Classification	Case I	Case II
Diabetic	1	Excluded
Undiagnosed diabetic	1	1
Prediabetic	0	1
Not diabetic	0	0

Case I - Records containing diabetic, pre / undiagnosed and non diabetic patients. Case II - Records containing pre / undiagnosed and non diabetic patients only. 1 - Positive record for the case; 0 - Negative record for the case (non diabetic patient)

**Table 3:** Cardiovascular disease classification criteria and label Assignments

Criteria		Classification	Label Assignment
Answered “yes” to having had one of the following ^*γ*^: congestive heart failure, coronary heart disease, heart attack, or stroke	⇒	Having heart diseases	1
If they answered “no” to all conditions	⇒	Not having heart diseases	0

*γ* - On the NHANES survey questionnaire. 1 - Positive record for CVD; 0 - Negative record for CVD

**Table 4:** The structure of the datasets used for diabetes and cardiovascular classification

Year	Case	Observations	Variables	No. of 0s	No. of 1s
1999-2014	Case I	21,131	123	15,599	5,532
1999-2014	Case II	16,426	123	9,944	6,482
2003-2014	Case I	16,443	168	11,977	4,466
2003-2014	Case II	12,636	168	7,503	5,133
2007-2014	Cardio	8,459	131	7,012	1,447

Case I and II datasets are for diabetes classification, Cardio dataset is for CVD classification. 1 - Positive records for the disease; 0 - Negative records for the disease

### Model Development

The datasets resulting from the aforementioned stage of Data Mining and Modeling (Section 4) were each split into training and testing datasets. Downsampling was used to produce a balanced 80/20 train/test split. In the training phase of the model development, the training dataset was used to generate learned models for prediction. In the validation phase, the models were tested with the features of the testing dataset to evaluate them on how well they predicted the corresponding class labels of the testing dataset. For each model, a grid-search approach with parallelized performance evaluation for model parameter tuning was used to generate the best model parameters. Next, each of the models underwent a 10-fold cross-validation (10 folds of training and testing with randomized data-split) in order to get an accurate measurement of model performance.

#### The Weighted Ensemble Model

For each individual model, the probability values of having the disease was recorded for each subject using a 10-fold cross-validation. Then a new probability was created for each subject from the weighted average of the probabilities from the individual models. This is the weighted ensemble model which uses a weighted average of individual model results. The weights were based on the performance of each model according to its AUC score. Suppose we label the four models (logistic regression, SVM, random forests, and gradient boosting) as models 1,2,3, and 4 respectively. The weight for the *i*th individual model was determined by

$$w_{i} = \frac{\text{AUC}_{i}^{2}}{\sum\nolimits_{i=1}^{4}\text{AUC}_{i}^{2}}. $$

The new probability for each subject was then calculated as

$$p_{new}=\sum\limits_{i=1}^{4} w_{i}p_{i}. $$

Each subject was then classified based on the new weighted probability.

#### Feature Selection

With a goal of creating an accurate model relying on a limited set of available features, i.e. features that did not require excessive questioning or testing of patients, we evaluated the feature dependence of the models for prediction of diabetes and CVD. The analysis was done based on the ensemble classifier of XGBoost (based on the model performance), where an error rate metric was used to rank features. More specifically, in XGBoost models, feature importance scores are calculated for each decision tree by how much the split-point(s) for each feature improves the binary classification error rate — weighted by the number of observations for which the split-point is responsible. The error rate is calculated as the number of mis-classified observations over the total number of observations. Finally, the importance scores are averaged over all trees in the model to create a final importance score for each feature [28].

The top 24 most important features were identified in each dataset. The cutoff of the 24 features was based on the cross-validation of the models, where lower than 24 features resulted in considerable drop in model performance (>2*%* AU-ROC score drop). The 24 features where then used to test other models, where no substantial drop in performance was recorded.

#### Performance Metrics

In the last stage of the pipeline illustrated in Fig. 1, the scores of the models were compared to evaluate their performance in predicting cases. The binary model evaluation (cases versus non-cases) was based on the performance statistics in terms of sensitivity ($\frac {TP}{TP+FP}$) and specificity ($\frac {TN}{TN+FN}$) where *TP*, *FP*, *TN*, and *FN* represent the number of true positives, false positives, true negatives, and false negatives, respectively. A false positive would be an observation that is predicted to be a case, but is not actually a case. A false negative can be defined similarly. Area under the curve (AUC) and receiver operating characteristic (ROC) were used to understand the relationship between the two performance variables. F1 scores were also used to measure a model’s accuracy; $F1 = 2\frac {precision*recall}{precision + recall}$ where $precision = \frac {TP}{TP + FP}$ and $recall = \frac {TP}{TP + FN}$. In other words, F1 score [29] is the harmonic average of the precision and recall allowing for comparison of different model performance in identifying true disease predictions when compared to false positives.

## Results

Table 5 describes the comparative accuracy scores of different models for diabetes prediction across different 1) cases (Case I and II), 2) timeframes, and 3) the type of feature variables (data with laboratory or only survey variables). As described in Section 4 (and shown in Table 4), each dataset has different number of observations and the variables used for the machine learning models.

**Table 5:** Results using 10-fold cross-validation for diabetes classification

Lab	Year & Case	Model	AUC	*Precision*	*Recall*	*F*1
No lab		Logistic Reg.	0.827	0.75	0.75	0.75
	1999-2014	SVM	0.849	0.77	0.77	0.77
	Diab. Case I	Random Forest	0.855	0.78	0.78	0.78
		**XGBoost**	**0.862**	**0.78**	**0.78**	**0.78**
		Ensemble	0.859	0.78	0.78	0.78
		Logistic Reg.	0.732	0.67	0.67	0.67
	1999-2014	SVM	0.734	0.68	0.68	0.68
	Diab. Case II	Random Forest	0.731	0.67	0.67	0.67
		XGBoost	0.734	0.67	0.67	0.67
		**Ensembl**e	**0.737**	**0.68**	**0.68**	**0.68**
		Logistic Reg.	0.800	0.72	0.72	0.72
	2003-2014	SVM	0.822	0.75	0.75	0.75
	Diab. Case I	**Random Forest**	**0.841**	**0.77**	**0.76**	**0.76**
		XGBoost	0.837	0.75	0.75	0.75
		Ensemble	0.834	0.75	0.75	0.75
		Logistic Reg.	0.718	0.66	0.66	0.66
	2003-2014	SVM	0.716	0.66	0.66	0.66
	Diab. Case II	Random Forest	0.719	0.67	0.67	0.66
		**XGBoost**	**0.725**	**0.67**	**0.67**	**0.67**
		Ensemble	0.725	0.66	0.66	0.66
With lab		Logistic Reg.	0.866	0.79	0.79	0.79
	1999-2014	SVM	0.887	0.81	0.81	0.81
	Diab. Case I	Random Forest	0.937	0.86	0.86	0.86
		**XGBoost**	**0.957**	**0.89**	**0.89**	**0.89**
		Ensemble	0.944	0.87	0.87	0.87
		Logistic Reg.	0.724	0.67	0.67	0.67
	1999-2014	SVM	0.737	0.68	0.68	0.68
	Diab. Case II	Random Forest	0.738	0.68	0.68	0.68
		**XGBoost**	**0.802**	**0.74**	**0.74**	**0.74**
		Ensemble	0.783	0.71	0.71	0.71
		Logistic Reg.	0.877	0.80	0.80	0.80
	2003-2014	SVM	0.882	0.81	0.80	0.80
	Diab. Case I	Random Forest	0.939	0.86	0.86	0.86
		**XGBoost**	**0.962**	**0.89**	**0.89**	**0.89**
		Ensemble	0.948	0.88	0.88	0.88
		Logistic Reg.	0.738	0.68	0.68	0.68
	2003-2014	SVM	0.737	0.68	0.68	0.68
	Diab. Case II	Random Forest	0.740	0.68	0.68	0.67
		**XGBoost**	**0.834**	**0.75**	**0.75**	**0.75**
		Ensemble	0.798	0.72	0.72	0.72

AUC - Area Under the Curve, $Precision = \frac {TP}{TP + FP}, Recall = \frac {TP}{TP + FN}$ (where *TP* - True Positive, *FP* - False Positive, *FN* - False Negative), and F1 (score) = $2\frac {precision*recall}{precision + recall}$. Bold face font signifies best performing model result

Within the time-frame of 1999-2014 for Case I diabetes prediction (data excluding laboratory results), the GBT based model of XGBoost (eXtreme Gradient Boosting) model performed the best among all classifier with an Area Under - Receiver Operating Characteristic (AU-ROC) of 86.2*%*. Precision, recall, and F1 scores were at 0.78 for all of the metrics using 10-fold cross validation of the model. The worst performing model in the class was linear model of Logistic Regression with an AU-ROC of 82.7*%*. Linear SVM model was close in performance to ensemble based models with an AU-ROC at 84.9*%*. Inclusion of laboratory results in Case I increased the predictive power of the models by a large margin, with XGBoost achieving a AU-ROC score of 95.7*%*. The precision, recall, and F1 scores were also recorded at 0.89 for the model.

In the prediction of prediabetic and undiagnosed diabetic patients – Case II (with the time-frame of 1999-2014), the developed Weighted Ensemble Model (WEM) has the top performance AU-ROC score of 73.7*%*. The recorded precision, recall, and F1-score were at 0.68. The WEM model was closely followed by other models Logistic Regression, SVM, RFC (Random Forest Classifier), and XGBoost each reporting an accuracy of 73.1−73.4*%* with 10-fold cross validation. The precision, recall, and F1-score scores were similar across the models. Case II performance analysis with the laboratory variables also results in a large performance increase to AU-ROC score of 80.2*%* in the timeframe of 1999-2014 and 83.4*%* in 2003-2014 timeframe, obtained by XGBoost in both cases.

**Fig. 2:**
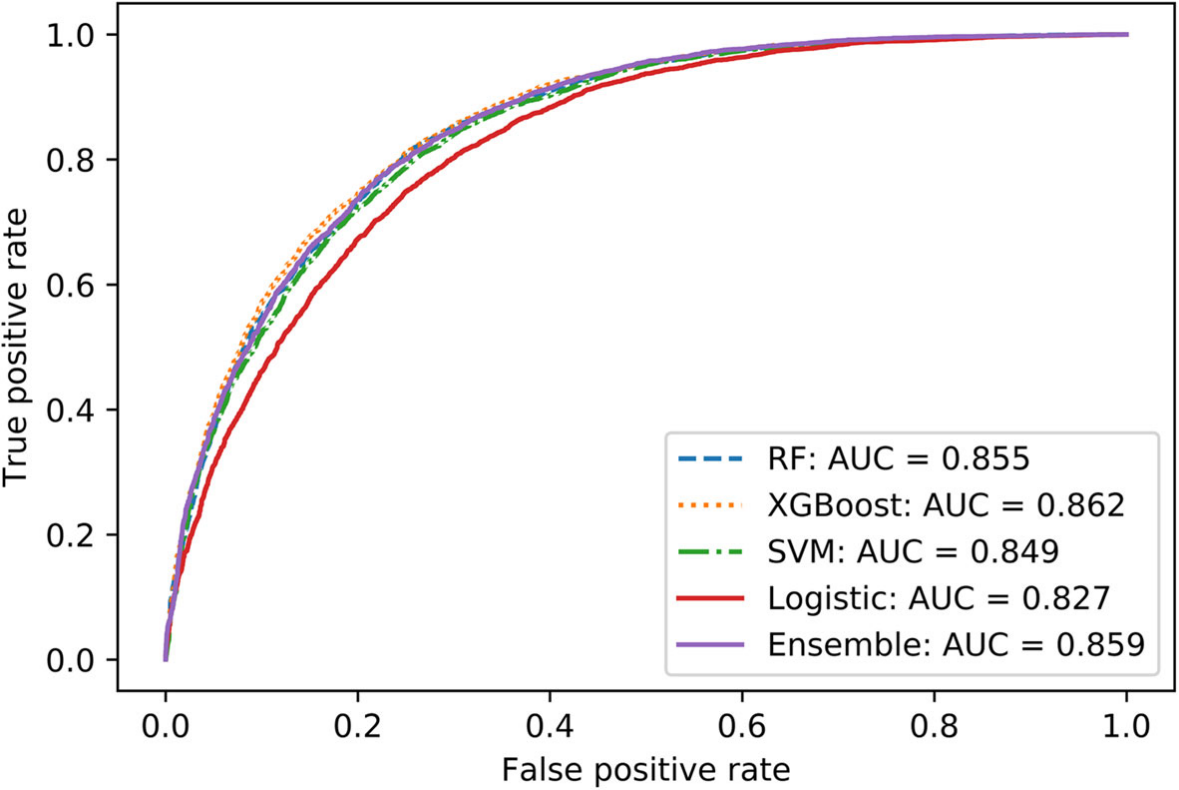
ROC curves from the 1999-2014 Diabetes Case I models. This graph shows the ROC curves generated from different models applied to the 1999-2014 Diabetes Case I datasets without lab

**Fig. 3:**
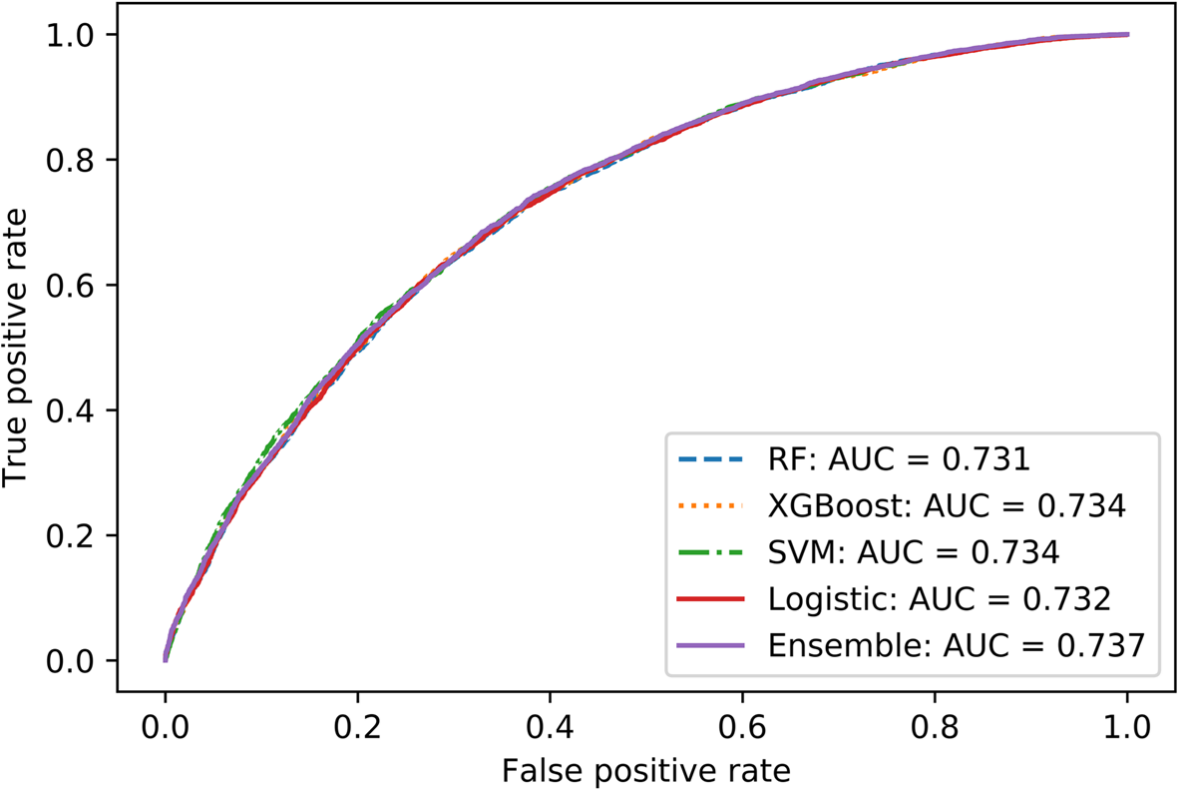
ROC curves from 1999-2014 Diabetes Case II models. This graph shows the ROC curves generated from different models applied to the 1999-2014 Diabetes Case II datasets without lab

Visualizing the model performance with receiver-operating characteristics (ROC), Figs. 2 and 3 shows the comparison of binary predictive power at various thresholds (false positive rate - FPR). The curves model the sensitivity — proportion of actual diabetic patients that were correctly identified as such, to the FPR or 1 - specificity, where specificity — proportion of non-diabetic patients that were correctly identified as such in the models. Analysis of models in Case I is shown in Fig. 2, and for Case II, Fig. 3 compares the performance of various models.

**Fig. 4:**
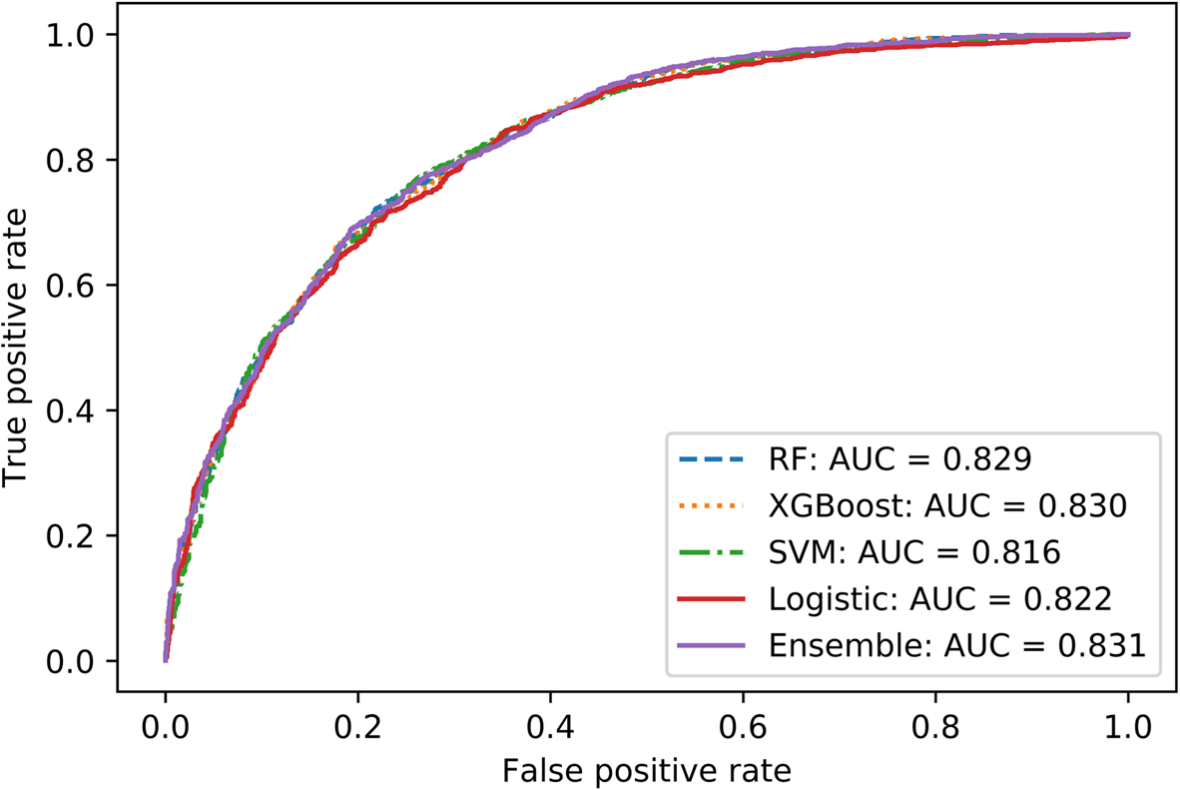
ROC curves from the cardiovascular models This graph shows the ROC curves generated from different models applied to the 1999-2007 cardiovascular disease datasets without lab

**Fig. 5:**
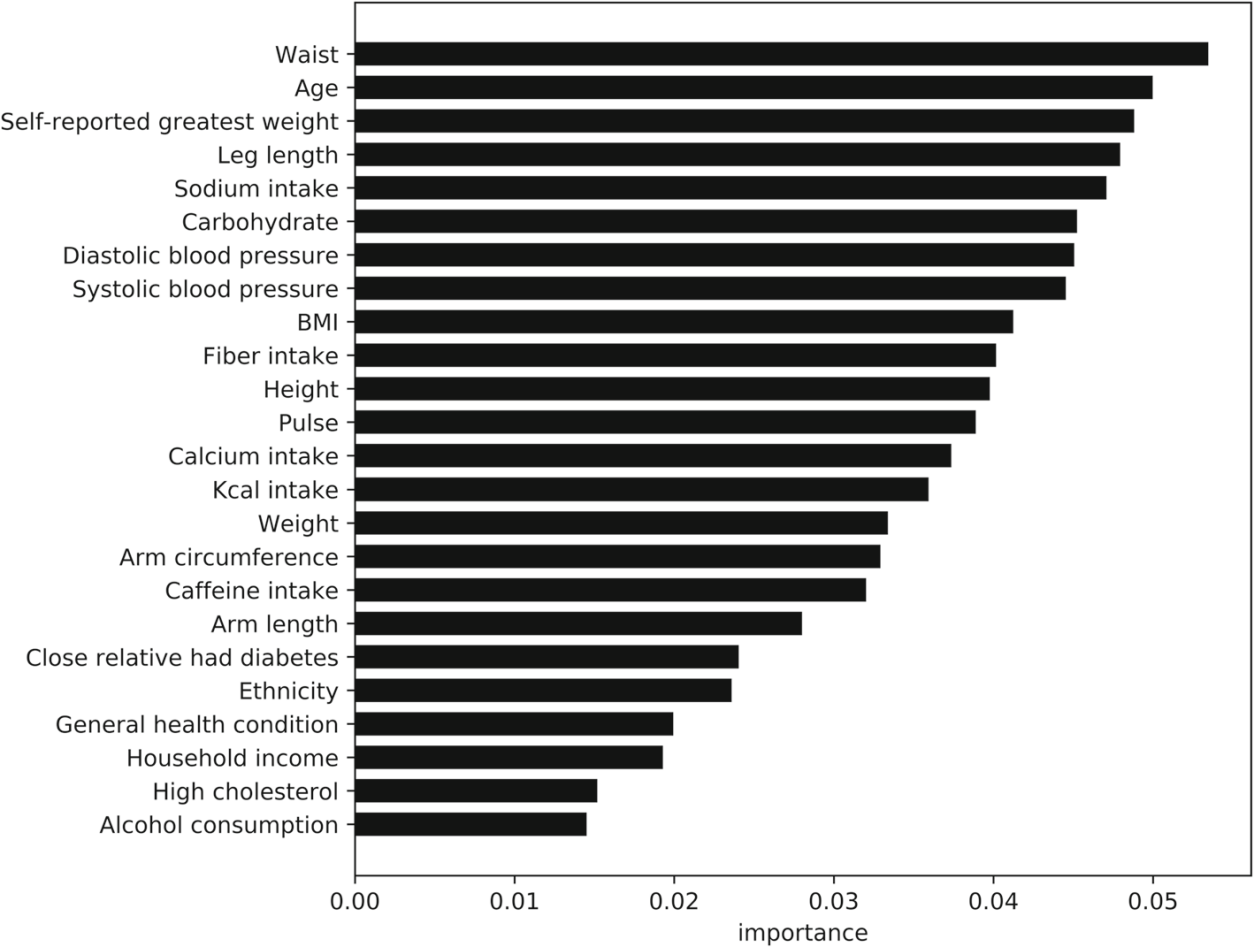
Average feature importance for diabetes classifiers without lab results. This graphs shows the most important features not including lab results for predicting diabetes

Using feature importance scores for the XGBoost model, Figs. 4 and 5 show the comparative importance of 24 variables/features in non-laboratory and laboratory based datasets for diabetes detection respectively. The results are based on the average error rate obtained by number of mis-classification of observations calculated over all sequential trees in an XGBoost classifier. The cut off of 24 features was obtained by developing models for each set of feature combinations (ordered by importance), and using a cutoff of ≤2*%* drop in the cross validation AU-ROC scores. The importance scores were also averaged for diabetic (Case I) and pre-diabetics/undiagnosed diabetic (Case II) models.

**Fig. 6:**
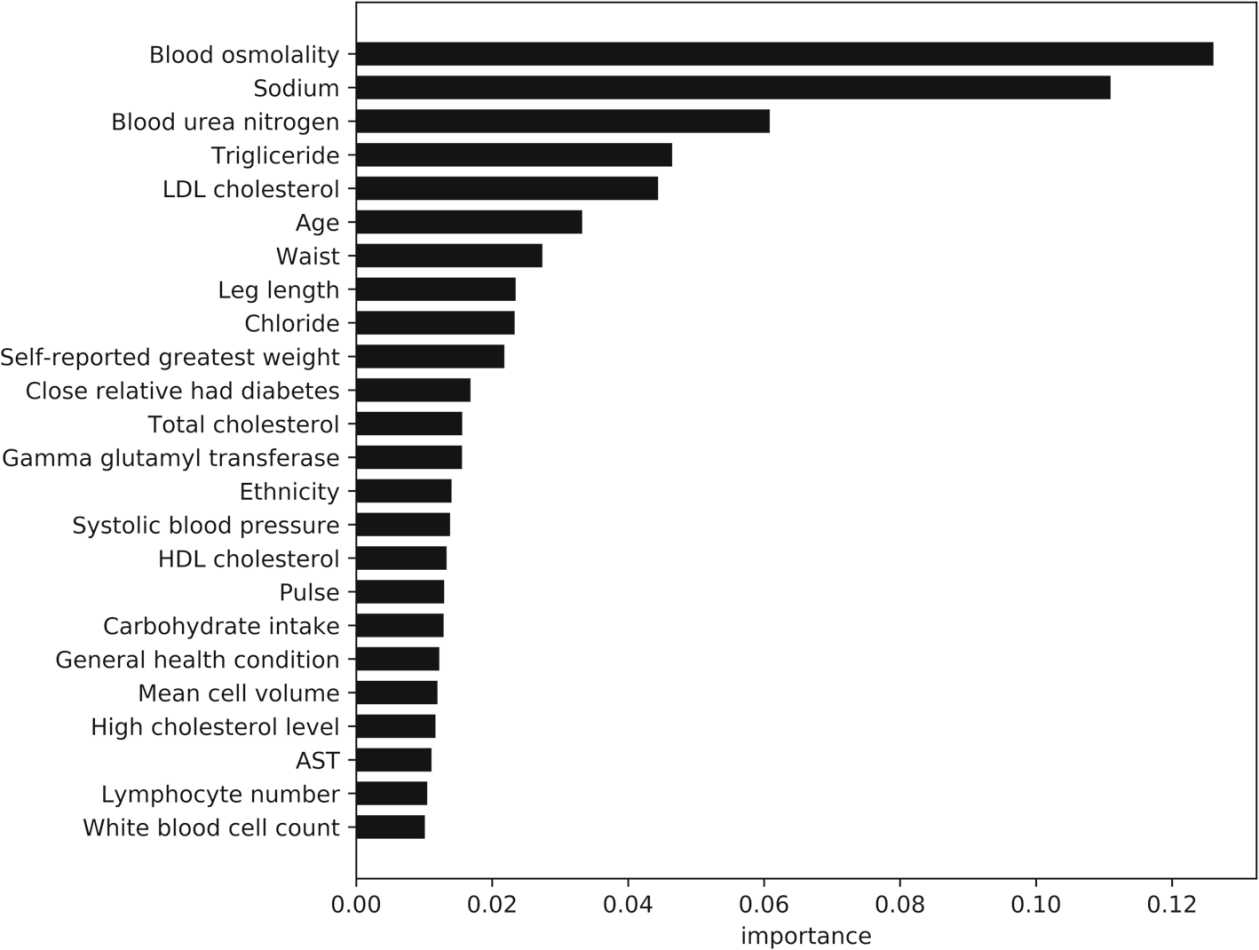
Average feature importance for diabetes classifiers with lab results. This graphs shows the most important features including lab results for predicting diabetes

Towards CVD classification, Table 6 compares the performance metrics of different models. Within the results, WEM performs the best with an AU-ROC score of 83.1*%* for non-laboratory data. Precision, recall, and F1-score of the model were pretty consistent at 0.75. Inclusion of laboratory based variables do not show any significant increase in performance, with an observed AU-ROC score of 83.9*%* obtained by the top performing WEM classifier. Performance metrics (Fig. 6) of different models — Logistic Regression, SVM, Random Forest, and WEM, shows similar accuracy scores recorded by all models (within 2% of AU-ROC score). Similar results are seen in the ROC curves for each of the models as shown in Fig. 6. While the ROC curve shows that the tree-based models - Random Forest and XGBoost (along with WEM) perform better than the other models, the difference is minimal.

**Table 6:** Results using 10-fold cross-validation for cardiovascular disease classification

Lab	Year	Model	AUC	*Precision*	*Recall*	*F*1
No lab		Logistic Reg.	0.822	0.74	0.74	0.74
	2007-2014	SVM	0.816	0.74	0.74	0.74
		Random Forest	0.829	0.75	0.74	0.74
		XGBoost	0.830	0.74	0.74	0.74
		**Ensemble**	**0.831**	**0.75**	**0.75**	**0.75**
With lab		Logistic Reg.	0.827	0.75	0.75	0.75
	2007-2014	SVM	0.825	0.75	0.75	0.75
		Random Forest	0.836	0.76	0.76	0.76
		XGBoost	0.838	0.76	0.76	0.76
		**Ensemble**	**0.839**	**0.76**	**0.76**	**0.76**

Lab - Laboratory results, AUC - Area Under the Curve, $Precision = \frac {TP}{TP + FP}, Recall = \frac {TP}{TP + FN}$ (where *TP* - True Positive, *FP* - False Positive, *FN* - False Negative), and F1 (score) = $2\frac {precision*recall}{precision + recall}$. Bold face font signifies best performing model result

**Fig. 7:**
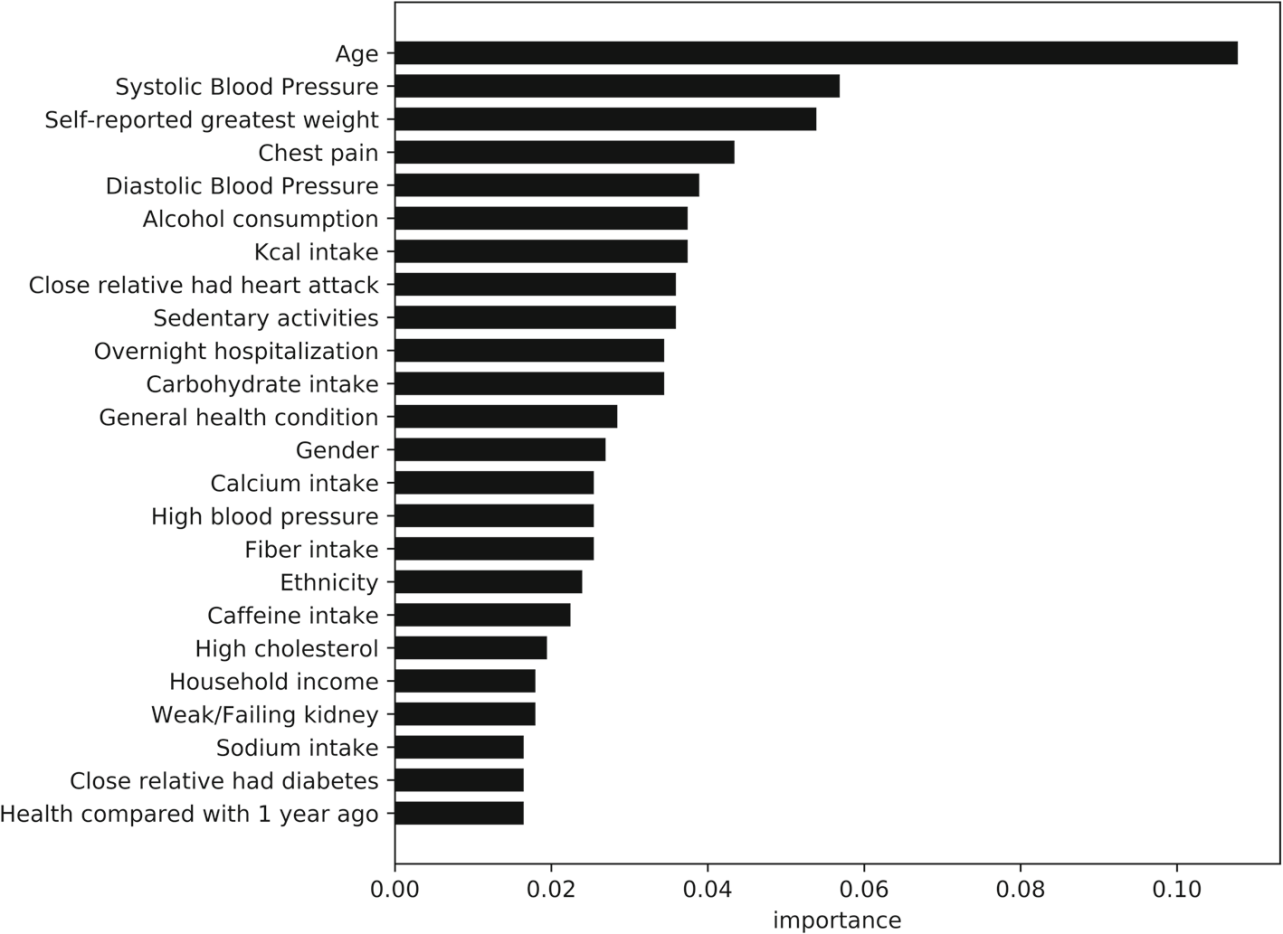
Feature importance for cardiovascular disease classifier without lab results This graphs shows the most important features not including lab results for predicting cardiovascular disease

**Fig. 8:**
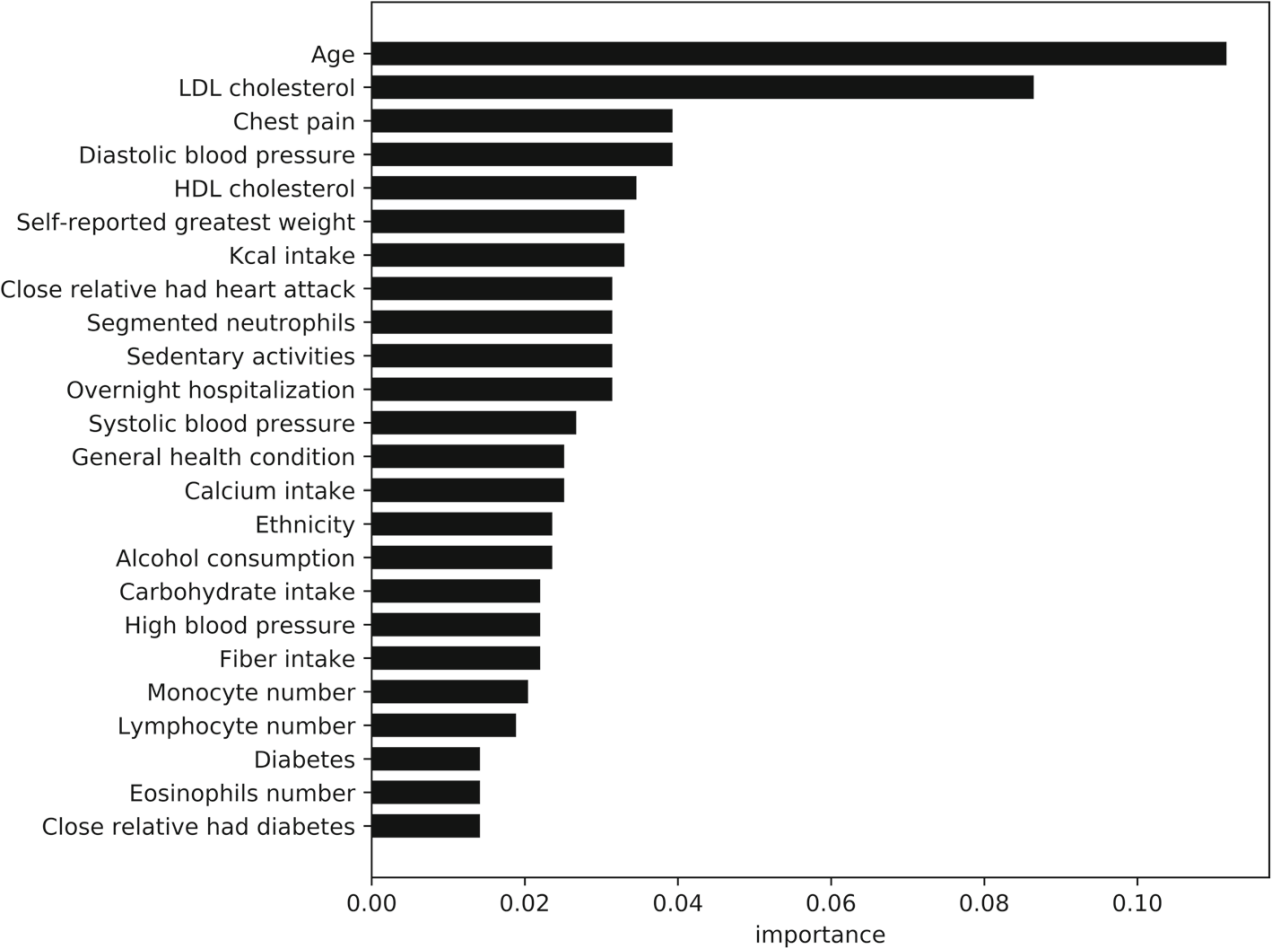
Feature importance for cardiovascular disease classifier with lab results This graphs shows the most important features including lab results for predicting cardiovascular disease

Figures 7 and 8, highlight the most important variables/features observed by the models trained on the non-laboratory and laboratory datasets respectively. As XGBoost was the top performing model in the category, information gain (based on error rate) was used to compare values between the variables within the model. Using similar approach to the diabetic analysis, average feature importance was measured with a cutoff at 24 variables.

## Discussion

### Diabetic Prediction

Models trained on diabetic patients (Case I) generally obtain a higher predictive power (86.2*%*) when compared to the Case II models which has a highest recorded accuracy of 73.7*%*. The decrease in detection performance in comparison to Case I is primarily due to two factors — 1) smaller number of observations, and 2) boundary conditions for the recorded observations. Case II only has 16,426 observations available in comparison to 21,091 observations available in Case I. The model also has difficulty in discerning fringe cases of patients, i.e. patients who are borderline diabetic versus normal. The accuracy also decreases slightly (AU-ROC at 72.5*%* for XGBoost) for the time-frame of 2003-2014, where there are even lower number of observations available for a larger number of variables. The consistency of the precision, recall, and F1 values suggests stable models with similar predictive power for diabetic (*label*=1) and non-diabetic (normal *label*=0) patients.

The WEM and XGBoost models developed in the study surpass prior research done by Yu et al. [13] where they obtained 83.5*%* (Case I) and 73.2*%* (Case II) using non-linear SVM models. While the number of observations and additional feature variables play a key part in the increased accuracy of our models, the ensemble based model consistently out-performed SVM in the diabetic study (especially for Case I). Comparing time-frames within our data, we observe for the window of 2003-2014 the best performing model (RFC) had a lower AU-ROC score was at 84.1*%* for Case I. While the timeframe has a larger set of features (168 versus 123), the drop in the number of observations (16,443 versus 21,091) leads to the reduction in accuracy by 2% when compared to 1999-2014. Similar results are also observed in Case II where the AU-ROC drops by 1.2*%* as a result of decrease in the number from 16,446 (in 1999-2014) to 12,636 (in 2003-2014).

Inclusion of laboratory results in Case I (1999-2014 timeframe) resulted in substantial increase the predictive capabilities (AU-ROC score of XGBoost - 95.7*%*). Contrary to previous observations, in the timeframe of 2003-2014, accuracy increases to 96.2*%* with XGBoost performing the best. This suggests availability of key laboratory variables within the 2003-2014 timeframe, leading to increased accuracy. Case II performance analysis with the laboratory variables also results in a large performance increase to AU-ROC score of 80.2*%* in the timeframe of 1999-2014 and 83.4*%* in 2003-2014 timeframe. XGBoost models perform the best in laboratory results in each of the cases, closely followed by the WEM model.

Model performance metrics for Case I shows tree based ensemble models - Random Forest and XGBoost along with the WEM model constantly outperform linear models such as Logistic Regression and Support Vector Machine. This is further highlighted in the ROC curves in Fig. 2. In Case II, the distinction is less obvious with similar performance recorded from all models as shown in Fig. 3. In such a case, computationally less demanding models such as Logistic Regression can be used to achieve similar classification performance when compared to other complex models such as SVM or ensemble classifiers.

Analysis of feature variables in non-laboratory based models (within the diabetes data) shows features such as waist size, age, weight (self-reported and actual), leg-length, blood-pressure, BMI, household income, etc. contribute substantially towards the prediction of the model. This is similar to the observations and variables used in prior research [12, 13]. However, in our study we observe several dietary variables such as sodium, carbohydrate, fiber, and calcium intake contribute heavily towards diabetes detection in our models. Caffeine and alcohol consumption, along with relatives with diabetes, ethnicity, reported health condition, and high cholesterol also play key roles. Within the laboratory based data, the feature importance measures suggest blood osmolality, blood urea nitrogen content, triglyceride, and LDL cholesterol are key factors in detection of diabetes. Each of the variables have been shown in prior research [30–33] to be key contributors or identifiers in diabetic patients. Age, waist circumference, leg length, weight, and sodium intake operate as common important variables for prediction between laboratory and survey data.

Prior research in the domain of predicting diabetes have reported results with high degree of accuracy. Using a neural network based approach to predict diabetes in the Pima Indian data set, Ayon et al. [34] observed an overall F1-score of 0.99. The analysis was based on data collected only from females of Pima Indian decent, and contained plasma glucose and serum insulin (which are key indicators of diabetes) as features for prediction. In comparison, our approach is a more generalized model where the demography of the patients is not restricted and does not contain plasma glucose and serum insulin levels (even in our laboratory based models). In [35] authors compare J48, AdaboostM1, SMO, Bayes Net, and Naïve Bayes, to identify diabetes based on non-invasive features. The study reports an F1 score of 0.95, and identify age as the most relevant feature in predicting diabetes, along with history of diabetes, work stress, BMI, salty food preferences, physical activity, hypertension, gender, and history of cardiovascular disease or stroke. While age, BMI, salt intake, and gender, were also identified in our study as pertinent variables, NHANES dataset does not contain (or has a high percentages of missing values) features of stress, history of cardiovascular disease, and physical activity. As a result the overall accuracy of the two studies cannot be compared directly. Heydari et al. [36] also compared SVM, artificial neural network (ANN), decision tree, nearest neighbors, and Bayesian networks, with ANN reporting the highest accuracy of 98%. However, study pre-screened for type 2 diabetes and was able to collect features of family history of diabetes, and prior occurrences of diabetes, gestational diabetes, high blood pressure, intake of drugs for high blood pressure, pregnancy and aborted pregnancy. Within our approach we consider both pre-diabetic and diabetic patients. Therefore, the results of this paper should be more accurate when applied to a diverse population which has not been screened for any pre-existing conditions.

### Cardiovascular (CVD) Prediction

Model performance towards the detection of at-risk patients of cardiovascular disease was pretty consistent across all models (AU-ROC difference of 1%, Fig. 6). While the WEM performed the best (AU-ROC 83.9*%*), other simplistic models such as logistic regression can provide similar results. This is partly due to the lack of large number of observations in the data, with total number of samples at 8,459, and also as a result of a high degree of imbalanced data with negative (0 label) versus positive (1 label) samples at 7,012 and 1,447 respectively. The applicability of ensemble based models (WEM, RFC, and XGBoost) can be further explored in the situations where large amounts of training observations are available, but in cases with limited observations computationally simple models like Logistic Regression can be used.

Models developed based on laboratory based variables do not show any significant performance gain with an increase of only 0.7*%*. This suggests a predictive model based on survey data only can provide an accurate automated approach towards detection of cardiovascular patients. Analyzing the features present in non-laboratory data, the most important features include age, diastolic and systolic blood pressure, self-reported greatest weight, chest pain, alcohol consumption, and family history of heart attacks among others. Incidents of chest pain, alcohol consumption, and family history of cardiac issues have been identified in prior research [37–39] as high risk factors for heart disease. As shown in study conducted by Lloyd-Jones et al. [40], age of the patients is a key risk variable in patients that is also identified by our models. A large number of feature importance variables are common across diabetes and cardiovascular patients, such as physical characteristics, dietary intake, and demographic characteristics. Similar factors (other than dietary variables) were identified by the study conducted by Stamler et al. [41], where they identified diabetes, age stratum, and ethnic background to be key contributors for cardiovascular disease.

The laboratory based data analysis suggests features such as age, LDL and HDL cholesterol, chest pain, diastolic and systolic blood-pressure, self-reported greatest weight, calorie intake, and family history of cardiovascular problems as important variables. LDL and HDL cholesterol have been shown as high risk factors of cardiovascular diseases in prior research [42, 43]. Segmented neutrophils, monocyte, lymphocyte and eosinophilis counts recorded in the laboratory variables also have importance in this classification model. Similar to non-laboratory results, dietary variables such as calorie, carbohydrate, and calcium intake reappear in the list of important features.

## Conclusion

Our study conducts an exhaustive search on NHANES data to develop a comparative analysis of machine-learning models on their performance towards detecting patients with cardiovascular and diabetic conditions. Compared to the Support Vector Machine based diabetic detection approach by Yu et al. [13], the models developed (based on non-laboratory variables) in our study show a small increase in accuracy (3% in Case I and 0.4*%* in Case II) achieved by the ensemble models - XGBoost and the Weighted Ensemble Model (WEM). Inclusion of laboratory based variables increase the accuracy of the learned models by 13% and 14% for Case I and II respectively. While laboratory based models do not present a realistic model, the features identified by the models can potentially be used to develop recommendation systems for at-risk patients.

The paper also explores the utility of such models on detection of patients with cardiovascular disease in survey datasets. Our study shows the machine-learned models based on WEM approach are able to achieve almost 84% accuracy in identifying patients with cardiovascular issues. We are also able to show models trained on only survey based responses perform almost at par with the data inclusive of laboratory results, suggesting a survey only based model can be very effective in detection of cardiovascular patients.

A key contribution of the study is the identification of features which contribute to the diseases. In diabetic patients, our models are able to identify the categories of — physical characteristics (age, waist size, leg length, etc.), dietary intake (sodium, fiber, and caffeine intake), and demographics (ethnicity and income) contribute to the disease classification. Patients with cardiovascular diseases are identified by the models based largely on their physical characteristics (age, blood pressure, weight, etc), issues with their health (chest pain and hospitalization incidents), and dietary (caloric, carbohydrate, fiber intake, etc.) attributes. A large set of common attributes exist between both diseases, suggesting that patients with diabetic issues may be also at risk of cardiovascular issues and vice-versa.

As shown in our analysis, machine learned models show promising results in detection of aforementioned diseases in patients. A possible real-world applicability of such a model can be in the form of a web-based tool, where a survey questionnaire can be used to assess the disease risk of participants. Based on the score, the participants can opt to conduct a more through check-up with a doctor. As a part of our future efforts, we also plan to explore the effectiveness of variables in electronic health records towards development of more accurate models.

## Data Availability

The National Health and Nutrition Examination Survey (NHANES) continuous data used in the study is available publicly at Center Disease Control (CDC) website at: https://www.cdc.gov/nchs/tutorials/nhanes/Preparing/Download/intro.htm. The documentation on how to download and use the data is provided at: https://www.cdc.gov/nchs/tutorials/NHANES/index_continuous.htm

## Abbreviations

AU-ROC: Area under - receiver operating characteristics
CDC: Center of disease control
GBT: Gradient boosted trees
NCHS: National center for health statistics
NHANES: National health and nutrition examination survey
RFC: Random forest classifier
SVM: Support vector machine
WEM: A weighted ensemble model
XGBoost: eXtreme gradient boosting
